# Pandemics, regional outbreaks, and sudden-onset disasters

**DOI:** 10.1177/0840470420901532

**Published:** 2020-02-05

**Authors:** Paul R. Gully

**Affiliations:** 1 The University of British Columbia School of Population and Public Health, Vancouver, British Columbia, Canada.

## Abstract

Pandemics of influenza, cholera, and plague are part of global history. Regional epidemics and pandemics of infectious diseases, primarily influenza A, continue to cause significant morbidity and mortality while remaining unpredictable in nature. Sudden-onset disasters such as earthquakes and floods occur with little warning. The consequences of climate change and environmental degradation can only be expected to increase the incidence of some infectious diseases and weather-related crises, adding to the unpredictability of such events. Health system leaders, both in public health and healthcare, need to understand the international context and how coordination and response across or within jurisdictions will improve the likelihood of successful management of challenges. Public health emergencies respect no borders or political structures. The ability of institutions to adapt quickly can make a difference in health outcomes and a community’s trust in those institutions.

## Introduction

The randomness of infectious disease events and sudden-onset disasters will continue to present challenges in detection and mitigation for the global community, including Canada. Through a review of recent events, this article highlights their unexpected nature and consequences and challenges in response. It is proposed that further efforts have to be made to improve the ability of the health systems of all countries to detect issues and to manage future challenges. Foreign policy and health action should be aligned to support global health.

## Recent events

### SARS and influenza

The Severe Acute Respiratory Syndrome (SARS) global outbreak began without warning in China late in 2002, gaining world attention in March 2003. SARS was readily transmissible by very sick people and produced severe disease. The causative agent, CoV, was quickly identified,^1,2^ as was the likely animal source and the means of spillover to humans,^3^ and diagnostic tests were widely available in a short space of time. The pattern of transmission allowed for the effective use of traditional public health measures but not before at least 8,096 cases had occurred globally, a significant proportion being in healthcare workers.

Canada was fully engaged in the response to SARS, although not without criticism, as examined in a report commissioned by the federal Minister of Health.^4^ Respect for the public health system and healthcare management in Canada was damaged. Institutions in Greater Toronto saw transmission of disease that caused tremendous health, social, economic, and political repercussions. The initial cases may have been unavoidable, but hospital management, infection control practices, and public health action should have been able to prevent the initial outbreak and the recrudescence. Chief Justice Campbell led the SARS Commission in Ontario^5^ and concluded that “we must ready for the unseen” (note 1).

Collaboration between governments, set up as part of pandemic influenza preparedness, was used to good effect, but it was evident that there needed to be radical improvements in surveillance and investigation capacity federally and provincially. The Public Health Agency of Canada was created in the wake of this event.

The difficulties of developed countries, including Canada, in recognizing and responding to SARS, led to acceptance by World Health Organization (WHO) Member States of revised International Health Regulations (IHR) for improved global management of outbreaks of infectious disease and other public health events.^6^

Although pandemic influenza preparedness plans were well advanced in many developed countries prior to late 2003, there was no concerted global effort to develop vaccines or plan to control global spread at source. The development of country preparedness had been stimulated by the outbreak of H5N1 in chickens and subsequent deaths in humans in Hong Kong in 1996. With its resurgence in late 2003, not long after the end of the SARS outbreak, there were predictions that H5N1 might give rise to a pandemic, but it was not until the influenza A (H1N1) pdm09 pandemic, in 2009, that there was the opportunity for a coordinated international response but plans were based on the assumption that an influenza pandemic would start in Asia. It was unexpected that a pandemic of human influenza would arise in a developed nation. Genetic analysis points to the origin being swine in Mexico.^7^ An increase in influenza-like illness was seen in that country in mid-March 2009, and swine influenza A/H1N1, similar to recent cases in California, was confirmed in Mexico by Canada, and by April, 28 cases were reported by WHO in Mexico, the United States, and Canada.^8^ The H1N1 pandemic was the first Public Health Emergency of International Concern declared under IHR (2005). Canada was able to respond with well-prepared plans, but there were still challenges in vaccine supply and delivery. An external review of the functioning of the IHR (2005) during the H1N1 pandemic concluded, “the world is (*still*) ill-prepared to respond to a severe influenza pandemic or to any similarly global, sustained and threatening public-health emergency.”^9^

### Middle East respiratory syndrome, Nipah, and Zika

Middle East respiratory syndrome also arose without warning and its provenance was unexpected. Middle East respiratory syndrome coronavirus (MERS-CoV) marks the second known zoonotic introduction of a highly pathogenic coronavirus, after SARS CoV, probably originating from bats.^10,11^ The virus has repeatedly spilled over from dromedary camels to humans, principally in countries on the Arabian Peninsula, causing significant morbidity and mortality. Clusters of cases in the community and families are rare, but nosocomial transmission occurs with large outbreaks in the Middle East and South Korea. As of August 2018, more than 2,249 cases from 27 countries had been reported to WHO.^12^ The novel animal source of the infection tested investigative abilities, and the unexpected outbreaks of nosocomial transmission strained detection and countries’ response capacities.

Nipah virus emerged, de novo, as a cause of illness and mortality in animals and humans and has persisted in Asia. The disease was first recognized in 1999 during an outbreak among pig farmers in Malaysia. It was then reported in Bangladesh in 2001 with subsequent outbreaks in Bangladesh and India and consumption of fruit or fruit products (such as raw date palm juice) contaminated with urine or saliva from fruit bats was found to be the most likely source of infection. Human-to-human transmission of Nipah virus has also been reported among family and caregivers of infected patients.^13^ SEARO (note 2) notes that the case fatality rate remained high during 2008-2012 despite a public awareness campaign and establishment of a referral system for better treatment in Bangladesh.^14^ Other regions may be at risk for infection, as evidence of the virus has been found in the natural reservoir (*Pteropus* bat species) and several other bat species in a number of countries, in Africa and Asia.

The outbreak of Zika virus–related disease in the Americas resulted from a change in geographic distribution of a virus recognized decades previously in another continent, but which re-emerged with devastating consequences. Cumulatively, there have been 27,647 reported cases from the Americas,^15^ and infection continues to carry the risk of Guillain-Barré syndrome and adverse pregnancy outcomes including increased risk of preterm birth, fetal death and stillbirth, and congenital malformations. Zika transmission has been found in all countries in the Americas except mainland Chile, Uruguay, and Canada.^16^ The data suggest that silent transmission among humans, animals, and mosquitoes has occurred throughout tropical Africa and Asia for more than 70 years, but that genetic changes in the virus resulted in emergence of a strain with increased transmissibility leading to greater epidemic potential and perhaps virulence.^17^ Brazil was the country initially affected and its response has been criticized, with suggestions that a previous lack of funding the public health system and healthcare facilities hampered the government’s response.^18^

### Ebola

Outbreaks of Ebola virus disease have been reported from Africa since 1972 when the disease was first recognized. It is therefore not new, but the magnitude and characteristics of recent outbreaks have severely pressured underfunded health systems. Between 2014 and 2016, a total of 28,610 confirmed, probable, and suspect cases were reported in Guinea, Liberia, and Sierra Leone, with 11,308 deaths.^19^ The challenges in control of transmission were complicated by social, cultural, and political factors, and the response of the international community and WHO, while well meaning, has been widely criticized.^20^ It was evident that, among other factors, the presence of a multitude of non-governmental and governmental actors was a significant challenge for national authorities.

The current outbreak of Ebola in the Democratic Republic of the Congo (DRC) began where outbreaks had been reported previously but where spread was limited. In 2019, widespread use of a vaccine would have been expected to halt transmission early but that has not happened. Enormous hurdles are presented by an absence of government control, the presence of militias and violence directed to healthcare workers, and pervasive suspicion of government and international efforts. The less well-known, concurrent, measles epidemic caused 2,750 casualties by mid-August. Médecins Sans Frontières (MSF) has drawn attention to the “contrast” between the Ebola response, where some funding has been quickly mobilized, and the measles response, where only $2.5 million have been received out of the $8.9 million needed.^21^

### Sudden-onset disasters

The Indian Ocean Tsunami of 2003 engendered a huge response from the global community, including Canada. It was evident in Indonesia that better management of responders was needed to make best use of the international effort. Whereas international guidelines for urban search and rescue operations were in place,^22^ this was not the case for emergency medical services. In the Nepal Earthquake of 2015, a WHO initiative developed during the West African Ebola outbreak in 2014-2015 was put in place enabling the government to ensure that medical teams arriving from outside of the country had the expertise, were self-sufficient, and operated under the command of the national authorities.^23^ The program recognizes the principle that the national government has ultimate authority over outbreak and disaster response.

The initial response to a disaster, recovery is often very long and is not necessarily a priority of the global community. It is interesting to note that even in a developed country such as New Zealand, recovery is prolonged. In Christchurch, the centre of that city only reopened 3 years after the earthquake in 2012, many buildings are still unstable and empty and reconstruction of some is not planned for years ahead.

## The international response

Infectious disease events and sudden-onset disasters will continue to challenge health systems. There have been important advances in response capacity, and IHR (2005) provides a binding framework that requires reporting and risk assessment of public health threats by Member States of WHO, as well as commitment to collaborate in capacity building. Non-government sources of data can now be used as a basis for informing WHO. Since coming into effect in 2007, IHR (2005) has democratized access to public health outbreak information and encouraged transparency by countries.

Efforts are being made by groups of WHO Member States, including Canada, to collaborate in various ways to improve response capacity.

The Global Health Security Initiative^24^ is an informal, international partnership among like-minded countries to strengthen health preparedness and response to threats of biological, chemical, radio-nuclear terrorism and pandemic influenza. Canada, the European Union, France, Germany, Italy, Japan, Mexico, the United Kingdom, and the United States launched this initiative in November 2001. During crisis situations, for example, during the 2014-2015 Ebola outbreak, regular teleconferences among Canada and partner countries allowed for the sharing of information regarding measures being adopted, informing national decision-making.

The Global Health Security Agenda was launched in February 2014 and is a growing partnership of over 64 nations, international organizations, and non-governmental stakeholders to help build countries’ capacity to “create a world safe and secure from infectious disease threats and elevate global health security as a national and global priority.”^25^

G7 Ministers of Health, meeting in 2019, stated that countries were “Making progress towards achieving Universal Health Coverage (UHC^26^[note 3]), global health security and pandemic and emergency preparedness and response…” and reiterated the commitment made by the G7 leaders in 2015 to offer assistance to countries to implement the WHO (IHR 2005).^27^

In 2018, the G20 Health Ministers reinforced “the need for joint commitment by G20 countries and the international community to strengthen core capacities for prevention: detection, preparedness, and response to emergencies within the context of health systems and the IHRs 2005.”^28^

Asian Pacific Economic Cooperation (APEC) and Economic Community of West African States (ECOWAS) have responded to threats such as SARS^29^ and Ebola.^30^

Current levels of support for WHO by Member States and other actions by countries are not enough (note 4). The Global Preparedness Monitoring Board of WHO 2019 Annual Report proposes seven urgent actions for leaders to take to prepare for national and global health threats including commitment to implementing binding obligations under the IHR (2005), countries and regional organizations including the G7, G20, and G77 leading by example.^31^

Less developed countries continue to struggle. The effectiveness of WHO remains limited and always dependent on continual efforts by staff to raise sufficient funds. The Emergency Committee of WHO, considering the Ebola Virus Disease (EBV) outbreak in DRC on October 18, 2019, heard of a lack of funding for preparedness, particularly for countries neighbouring DRC. Of the $66.6 million required for these nine countries, only $4.5 million has been pledged. The World Bank has concluded “most countries would need to spend just $1-2 per person per year to reach an acceptable level of health emergency preparedness. That amounts to a return on investment of ten to one, or even higher. And the return on investment does not consider the benefits beyond health for the economy or social stability. In today’s deeply interconnected world, if one community cannot prevent or manage disease outbreaks, everyone is at risk.”^32^

## Foreign policy and global health

Whether we achieve further successes in global health or our efforts are undermined by the pursuit of traditional foreign-policy interests, will depend on the ability of public health practitioners to understand foreign-policy perspectives on health and promote global health interests in the world of high politics.^33^

There is an important connection between global health security^34^ and a country’s foreign policy. Foreign policy will influence decisions to act to promote health security in an outbreak which can aid control of the disease for the good of the people of the affected country and/or can have the objective of reducing the risk of spread to the provider country. Foreign states may decide to intervene to prevent disease and disaster-associated conflicts from enabling insurgents, regional spread and to reduce peace-keeping costs.^35^

The involvement of donor countries may reflect past colonial interest and maintenance of sphere of influence. This was clear, for example, in the outbreak of Ebola in West Africa where the UK’s efforts were directed to Sierra Leone and those of the United States of America to Liberia. Canada supported all three countries involved including Guinea, another member of Organisation internationale de la Francophonie. David Fidler, a legal scholar who has been directly involved in WHO’s efforts on the IHR (2005) stated that “When diseases threaten, or show the potential to threaten, national security, military capabilities, geopolitical or regional stability, national populations, economic power, and trade interests, foreign policy-makers take notice.”^36^

Foreign policy or a country’s investments could have a negative influence on an outbreak. The present outbreak of Ebola in DRC is in areas where the extraction of the so-called conflict minerals and metallic ores including gold and coltan is an important source of revenue for government and militias. Insurgents have been implicated in direct attacks on healthcare workers and resistance to control measures including immunization is related to anti-government movements and antagonism to foreign workers suspected of spreading the disease intentionally. Canadians and the Government of Canada should be aware of, and examine, the business interests of Canadian-owned companies to see if they are negatively influencing the political and consequently the health situation. This could be a role for the newly appointed Ombudsperson for responsible Enterprise.^37^ Coltan, gold, diamonds, and other minerals may not be the only sources of funding for the rival militias and armies, but they are significant components of financing. According to one commentator, the G-20, and Organisation for Economic Cooperation and Development (OECD), countries should immediately scrutinize the conflict minerals trade and attempts to limit their use in cell phones and other electronics.^38^

## The national and local context

Outbreaks of infectious diseases that extend across international borders may be of direct significance to health system leaders. Managers need ready access to up-to-date risk assessments relevant to their responsibilities. The WHO Health Emergency Dashboard^39^ gives a global perspective but, in Canada, information should also be available through PHAC,^40^ provincial agencies such as the British Columbia Centre for Disease Control,^41^ Institut national de santé publique of the Province of Quebec,^42^ Public Health Ontario,^43^ and local public health authorities. Assessments of the risk of sudden-onset disasters should be available through provincial agencies responsible for emergency response. If assessments important to health managers are not routinely available and accessible, it is suggested that this be on the agenda of discussions between federal, provincial, and territorial health leaders.

## Conclusions

Member states of WHO should continue to examine their own health system’s capacity to respond to emergencies but also to assist less developed countries to develop capacity to confront outbreaks and sudden-onset disasters.

Countries and the global community will always have to deal with novel threats. The speed and effectiveness of response can be improved for the benefit of affected countries, regions, and the world, but this will require increased funding for IHR (2005)-related capacity building as well as an enlightened response by developed countries beyond immediate health and national security concerns toward long-term rebuilding of a country’s infrastructure. Countries should also ensure that their foreign policies do not hinder disease control and disaster response efforts but are used to actively support such efforts. Canada’s public health, emergency preparedness, and healthcare systems should be highly interconnected to ensure appropriate and timely emergency responses to international, national, and local threats.

## Notes

1. “SARS taught us that we must be ready for the unseen. That is one of the most important lessons of SARS. Although no one did foresee and perhaps no one could foresee the unique convergence of factors…that made SARS a perfect storm, we know now that new microbial threats like SARS have happened and can happen again. However, there is no longer any excuse for governments and hospitals to be caught off guard and no longer any excuse for health workers not to have available the maximum level of protection through appropriate equipment and training.”

2. SEARO is the South East Asian Regional Office of WHO.

3. Universal health coverage includes health security.

4. Canada is the ninth largest donor country, spending $4.7 billion on total Official Development Assistance (ODA) in 2018. This represents 0.28% of its Gross National Income (GNI) which is far below the United Nations target of 0.7% but for Canada. ODA on global health as a percentage of all ODA was 19.2% in 2016, second only to the United States.
